# The Roles of Sphingosine Kinase 1 and 2 in Regulating the Metabolome and Survival of Prostate Cancer Cells

**DOI:** 10.3390/biom3020316

**Published:** 2013-06-10

**Authors:** Francesca Tonelli, Manal Alossaimi, Viswanathan Natarajan, Irina Gorshkova, Evgeny Berdyshev, Robert Bittman, David G. Watson, Susan Pyne, Nigel J Pyne

**Affiliations:** 1 Cell Biology Group and Pharmaceutical Analysis and Metabolomics Research Group, Strathclyde Institute of Pharmacy and Biomedical Sciences, University of Strathclyde, 161 Cathedral Street, Glasgow G4 0RE, UK; E-Mails: f.tonelli@dundee.ac.uk (F.T.); manal.alossaimi@strath.ac.uk (M.A.O.); d.g.watson@strath.ac.uk (D.G.W.); susan.pyne@strath.ac.uk (S.P.); 2 University of Illinois at Chicago Department of Pharmacology, 909 S. Wolcott Ave., Chicago, IL 60612, USA; E-Mail: visnatar@uic.edu; 3 University of Illinois at Chicago, Department of Medicine, 909 S. Wolcott Ave., Chicago, IL 60612, USA; E-Mails: irgorshko@uic.edu (I.G.); eberdysh@uic.edu (E.B.); 4 Department of Chemistry and Biochemistry, Queens College of the City University of New York, Flushing, New York 11367-1597, USA; E-Mail: robert.bittman@qc.cuny.edu (R.B.)

**Keywords:** Sphingosine kinase inhibitors, lyso-phosphatidylinositol, sphingolipids, diadenosine 5′,5′′′-P^1^,P^3^-triphosphate

## Abstract

We have previously shown that treatment of androgen-sensitive LNCaP cells with the sphingosine kinase (SK) inhibitor SKi (2-(*p*-hydroxyanilino)-4-(*p*-chlorophenyl)thiazole) induces the proteasomal degradation of two N-terminal variants of SK1 (SK1a and SK1b), increases C22:0-ceramide and diadenosine 5′,5′′′-P^1^,P^3^-triphosphate (Ap3A) and reduces S1P levels, and promotes apoptosis. We have now investigated the effects of three SK inhibitors (SKi, (*S*)-FTY720 vinylphosphonate, and (*R*)-FTY720 methyl ether) on metabolite and sphingolipid levels in androgen-sensitive LNCaP and androgen-independent LNCaP-AI prostate cancer cells. The 51 kDa N-terminal variant of SK1 (SK1b) evades the proteasome in LNCaP-AI cells, and these cells do not exhibit an increase in C22:0-ceramide or Ap3A levels and do not undergo apoptosis in response to SKi. In contrast, the SK inhibitor (*S*)-FTY720 vinylphosphonate induces degradation of SK1b in LNCaP-AI, but not in LNCaP cells. In LNCaP-AI cells, (*S*)-FTY720 vinylphosphonate induces a small increase in C16:0-ceramide levels and cleavage of polyADPribose polymerase (indicative of apoptosis). Surprisingly, the level of S1P is increased by 7.8- and 12.8-fold in LNCaP and LNCaP-AI cells, respectively, on treatment with (*S*)-FTY720 vinylphosphonate. Finally, treatment of androgen-sensitive LNCaP cells with the SK2-selective inhibitor (*R*)-FTY720 methyl ether increases lysophosphatidylinositol levels, suggesting that SK2 may regulate lyso-PI metabolism in prostate cancer cells.

## 1. Introduction

Sphingosine 1-phosphate (S1P) is a bioactive lipid that has an important role in both solid tumors and hematological cancers [1]. S1P binds to 5 cell-surface G-protein coupled S1P receptors (termed S1P_1–5_) to induce a multitude of cell responses [1]. S1P binds to intracellular targets such as histone deacetylases 1 and 2 [2,3,4]. Two isoforms of sphingosine kinase, SK1 and SK2, catalyze the formation of S1P in mammalian cells. SK1 and SK2 are encoded by different genes and have unique biochemical properties and different subcellular localization and function [1]. There is substantial evidence that SK1 is involved in cancer; for example, SK1 mRNA transcript and/or protein expression are increased in various human tumors [1]. Moreover, large vascularized resistant tumors are formed when cancer cells over-expressing SK1 are injected or implanted into mice [1,4]. There is also evidence for an important role of SK2 in cancer. For instance, siRNA knockdown of SK2 enhances doxorubicin-induced apoptosis of breast or colon cancer cells [5] and reduces cancer cell proliferation and migration/invasion [6]. 

LNCaP prostate cancer cells express two N-terminal variant isoforms of SK1: SK1a (GenBank number: NM_001142601), which is a 42.5-kDa protein, and SK1b (GenBank number: NM_182965), which is a 51-kDa protein identical to SK1a but has an 86 amino acid N-terminal extension (sometimes referred to as SK1c). We have previously reported that the treatment with SK inhibitor SKi (2-(*p*-hydroxyanilino)-4-(*p*-chlorophenyl)thiazole) for 24–48 h activates the proteasome to accelerate the degradation of SK1a and SK1b in androgen-sensitive LNCaP prostate cancer cells [7,8]. This leads to a reduction in the intracellular S1P level and an elevation of sphingosine and C22:0-ceramide levels, which is concomitant with apoptosis [7,9,10]. However, SK1b evades the proteasome in androgen-independent LNCaP-AI prostate cancer cells and SKi fails to induce apoptosis of these cells [7]. This is also associated with failure of SKi to increase ceramide levels in LNCaP-AI cells [9]. FTY720 is a SK1 inhibitor and also induces proteasomal degradation of SK1 in MCF-7 breast cancer and LNCaP prostate cancer cells [8]. 

Small changes in the structure of the FTY720 scaffold have a marked effect on the nature of the inhibition of SK1. For instance, FTY720 is a competitive inhibitor (with sphingosine), while (*S*)-FTY720 vinylphosphonate is an uncompetitive inhibitor with sphingosine and a mixed inhibitor with ATP [9,11]. Moreover, (*S*)-FTY720 vinylphosphonate induces proteasomal degradation of SK1a and SK1b in androgen-independent LNCaP-AI prostate cancer, thereby inducing apoptosis of these cells [8]. 

We have also shown that several glycolytic metabolites and *(R)-*S-lactoylglutathione, an intermediate of methylglyoxal degradation and an anti-proliferative compound [12], are increased upon treatment of androgen-sensitive LNCaP cells with SKi, which acts predominantly on SK1 rather than SK2 in these cells [10]. These changes reflect an indirect antagonism of the Warburg effect. LNCaP cells also respond to SKi by diverting glucose 6-phosphate to the pentose phosphate pathway to provide NADPH, which serves as an antioxidant to counter an oxidative stress response [10]. SKi also promotes the formation of a novel pro-apoptotic molecule called diadenosine 5′,5′′′-P^1^,P^3^-triphosphate (Ap3A) in LNCaP cells, which binds to the tumor suppressor fragile histidine triad protein (FHIT), suggesting a link between SK1 and FHIT signaling [10]. 

We have investigated the role of SK2 in cancer using selective SK2 inhibitors. (*R*)-FTY720-OMe (ROME) is a selective, enantioselective, competitive (with sphingosine) inhibitor of SK2 [13]. Treatment of breast MCF-7 cancer cells with ROME prevents actin enrichment into lamellipodia in response to S1P, suggesting that metastasis can be inhibited [13]. In addition, DNA synthesis in MCF-7 cells is reduced by ROME [13]. The K_i_ value for inhibition of SK2 activity by ROME is 16.5 μM; thus, ROME is similar in potency to another SK2-selective inhibitor, ABC294640, which has a K_i_ of 10 μM [14]. Treatment of androgen-sensitive LNCaP cells with ROME reduces glycolytic metabolite levels and does not induce oxidative stress [10]. In addition, ROME activates autophagy and has no effect at low concentrations (that inhibit SK2) on AR expression [10], while SKi inhibits autophagy and reduces AR expression in LNCaP cells [10,15]. These findings suggest that SK2 has an opposing action to SK1 in androgen-sensitive LNCaP cells. 

In the current study we have investigated the effect of the SK inhibitor SKi on the metabolome of androgen-independent LNCaP-AI cells. These studies demonstrate that Ap3A is not formed in response to SKi in these cells. As stated earlier, the resistance of SK1b to proteasomal degradation in androgen-independent LNCaP-AI cells in response to SKi can be circumvented by the use of the SK inhibitor, (*S*)-FTY720 vinylphosphonate [8]. We have, therefore, also assessed the effect of (*S*)-FTY720 vinylphosphonate on sphingolipid and metabolite levels to provide information about how it induces apoptosis of LNCaP-AI cells. In addition, we have analyzed the effect of the SK2-selective inhibitor ROME on the metabolome to reveal further differences in the functional roles of SK1 and SK2 in LNCaP cells. Inhibition of SK2 in LNCaP cells by ROME resulted in a marked elevation of 1-palmitoyl-2-lyso-*sn*-3 glycerophosphoinositol (>200 fold), providing the first evidence for a role of SK2 in the regulation of lyso-glycerophospholipid metabolism. 

## 2. Results and Discussion

### 2.1. Effect of SKi on the Metabolome of LNCaP-AI Cells

In order to assess why SK1b protects LNCaP-AI cells from SKi-induced apoptosis we established the effect of SKi on the metabolome of LNCaP-AI cells. The metabolites listed in Table 1 were identified according to their accurate masses, which all had <2 ppm mass deviation from the exact mass of the specific metabolite. The metabolite database was used as a filter to exclude any other possible metabolites according to the metabolic standards initiative [16]. Common isomers of metabolites were differentiated by matching the retention times with those of isomeric standards. We found that the treatment of LNCaP-AI cells with SKi (10 μM, 24 h) increased the levels of glycolytic metabolites that include fructose 1,6-bisphosphate, D-glyceraldehyde 3-phosphate, dihydroxyacetone phosphate, 3-phosphoglycerate, and (*S*)-lactoylglutathione (a byproduct of the glycolytic pathway that accumulates from dihydroxyacetone) (Table 1). These effects reflect antagonism of aerobic glycolysis (the Warburg effect) by SKi. LNCaP-AI cells also respond to SKi by diverting glucose 6-phosphate into the pentose phosphate pathway in order to provide NADPH to counter oxidative stress responses (Table 1). The glutathione (GSH) system uses NADPH to reduce the oxidized form, GSSG, to GSH. The levels of GSSG and pentose phosphate pathway intermediates (ribulose 5-phosphate and phosphogluconate) are increased and the NADPH level is reduced by SKi (Table 1). These changes are also common to androgen-sensitive LNCaP cells treated with SKi (10). In contrast to LNCaP cells, LNCaP-AI cells do not undergo apoptosis in response to SKi (7). A major difference between the two cell types is the absence of Ap3A in LNCaP-AI cells (Ap3A levels in LNCaP cells: peak height; control, 1776 +/− 18.9%; SKi-treated, 3809 +/− 10.3%, n = 6). The absence of Ap3A in LNCaP-AI cells is associated with higher SK1a and SK1b expression levels in these cells compared with LNCaP cells [8] and an ability of SK1b to evade the proteasome in response to SKi [7].

**Table 1:** Effect of SKi (10 μM, 24 h) on metabolite levels in LNCaP-AI cells. Results are expressed as the ratio SKi:control for each metabolite. P values are presented for SKi *vs.* control (experimental results from n = 6 cell samples for each treatment).

	Ratio of SKi : control	P value n = 6
**Oxidative Stress**		
GSSG	12.4	<0.001
NADP^+ ^	5.9	<0.001
NADPH	0.24	<0.0001
Phosphogluconate	4.4	<0.05
Ribulose phosphate	3.7	<0.01
**Glycolysis/TCA cycle**		
Dihydroxyacetone phosphate	7.6	<0.005
D-Glyceraldehyde 3-phosphate	23.4	<0.01
3-Phosphoglycerate	2.7	<0.05
Fructose 1,6-bisphosphate	5.2	<0.0001
S-D-Lactoylglutathione	28.3	<0.001

Since FHIT binds to Ap3A and induces apoptosis [17], the presence or absence of Ap3A in SKi-treated androgen-sensitive LNCaP cells and androgen-independent LNCaP-AI cells, respectively, might provide an explanation for the different sensitivities of these cells to the apoptotic effect of SKi. Therefore, our findings suggest that Ap3A might cooperate with the antagonism of the Warburg effect and oxidative stress to induce apoptosis of androgen-sensitive LNCaP cells. Thus the absence of Ap3A in SKi-treated androgen-independent LNCaP-AI cells might enable these cells to mitigate the oxidative stress and antagonism of the Warburg effect, thereby escaping the apoptotic program. These results are also relevant to prostate cancer biology as intragenic alterations in the FRA3B/FHIT chromosome fragile site results in fragile FHIT allele loss early in cancer development [17]. Moreover, *Fhit* knockout mice are also predisposed to tumor development and Fhit gene therapy reduces tumor burden [18] and restoration of wild-type FHIT in 3p14.2-deficient human lung cancer cells also inhibits cell growth and induces apoptosis [19].

### 2.2. Effect of (S)-FTY720 Vinylphosphonate on Sphingolipids in LNCaP and LNCaP-AI Cells

**Figure 1:**
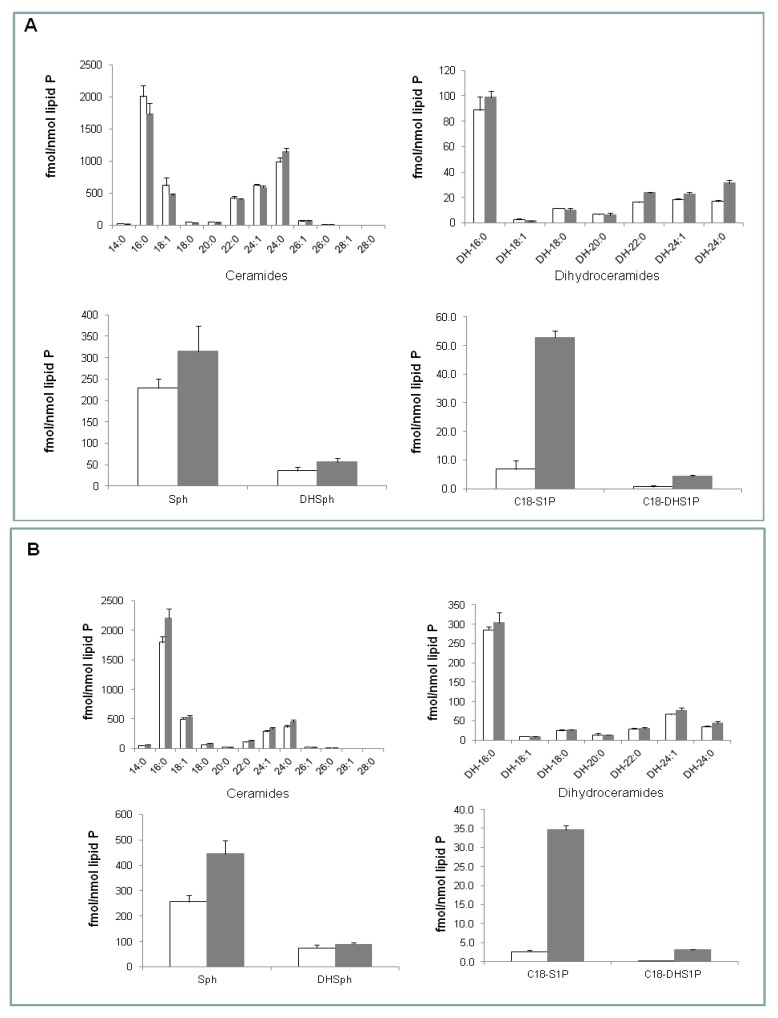
(**A**) Effect of (*S*)-FTY720 vinylphosphonate on sphingolipid levels in LNCaP cells. P values for treated versus control, DH 22:0, 24:1 and 24:0, *p* < 0.01; C18-DHS1P, *p* < 0.01; S1P, *p* < 0.001; (**B**) Effect of (*S*)-FTY720 vinylphosphonate on sphingolipid levels in LNCaP-AI cells. P values for treated versus control, C16:0 ceramide, *p* < 0.02; sphingosine, *p* < 0.01; DHS1P or S1P, *p* < 0.001. In both cases, cells were treated with (*S*)-FTY720 vinylphosphonate (10 μM, 24 h, shaded bar) or with vehicle (DMSO, 0.1% v/v, 24 h, open bar) alone and samples subjected to LC-MS/MS analysis. Results are means +/− SD for n = 3 samples.

**Figure 2:**
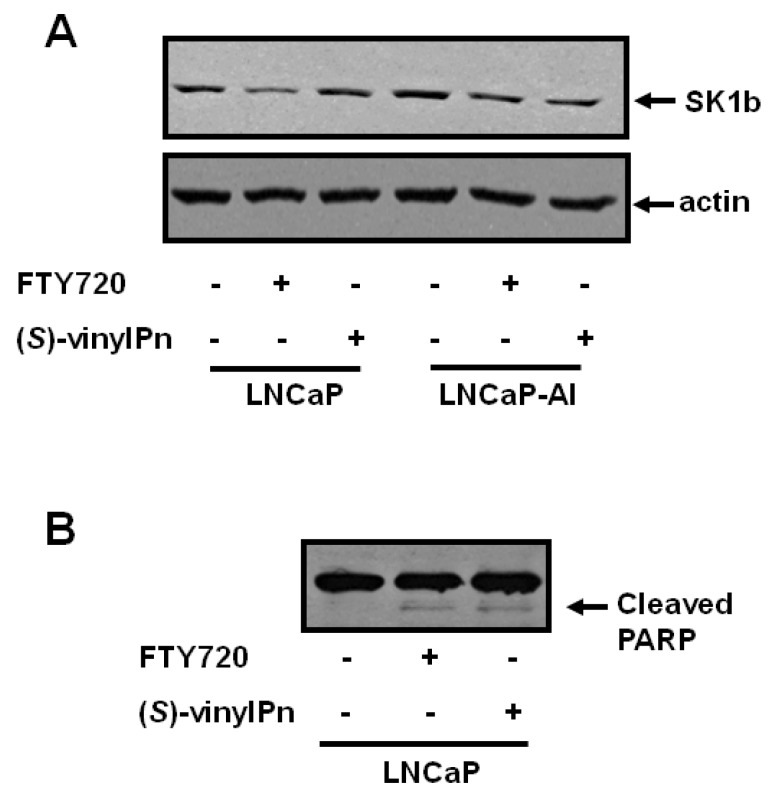
Effect of (*S*)-FTY720 vinylphosphonate on SK1b expression and PARP cleavage in LNCaP and LNCaP-AI cells. LNCaP cells or LNCaP-AI cells were treated with FTY720 (10 μM, 48 h, for comparison), (*S*)-FTY720 vinylphosphonate (10 μM, 48 h), or with vehicle (DMSO, 0.1% v/v, 48 h) alone. The lysates were subjected to western blot analysis with (**A**) anti-SK1b antibody (for comparison the effect of (*S*)-FTY720 vinylphosphonate and FTY720 on SK1b expression is shown in LNCaP-AI cells), or (**B**) anti-PARP antibody. Results are representative of at least 3 separate experiments. (*S*)-vinylPn represents (*S*)-FTY720 vinylphosphonate.

The ability of SK1b to evade the proteasome in response to SKi in androgen-independent LNCaP-AI cells can be circumvented by the use of the SK1 selective inhibitor (*S*)-FTY720 vinylphosphonate [8]. This inhibitor induces the proteasomal degradation of both SK1a and SK1b and promotes apoptosis of LNCaP-AI cells [8]. We have, therefore, assessed the effect of (*S*)-FTY720 vinylphosphonate on sphingolipid levels in both LNCaP and LNCaP-AI cells. Using electrospray ionization tandem mass spectrometry, we demonstrate that (*S*)-FTY720 vinylphosphonate induces a small but significant increase in the levels of long-chain dihydroceramides (C22:0-, C24:0-, and C24:1-dihydroceramides), and surprisingly elicited a dramatic increase (7.8 fold) in the sphingosine 1-phosphate (S1P) level and a small increase in the dihydrosphingosine 1-phosphate (DHS1P) level in LNCaP cells (Figure 1A). Notably, (*S*)-FTY720 vinylphosphonate did not affect the levels of various molecular species of ceramide in LNCaP cells (Figure 1A). The small increase in dihydroceramides in LNCaP cells might reflect activation of the *de-novo* ceramide synthesis pathway in response to (*S*)-FTY720 vinylphosphonate. Notably, (*S*)-FTY720 vinylphosphonate did not reduce expression of SK1b in LNCaP cells (Figure 2A). 

Treatment of LNCaP-AI cells with (*S*)-FTY720 vinylphosphonate significantly increased the sphingosine level and also produced a small increase in the C16:0-ceramide level (control, 1799 +/− 96; (*S*)-FTY720 vinylphosphonate, 2213 +/− 159 fmol/nmol lipid phosphorous, results expressed as means +/− SD, n = 3), but had no effect on dihydroceramide levels (Figure 1B). (*S*)-FTY720 vinylphosphonate also induced a dramatic increase (12.8 fold) in S1P level and a small increase in DHS1P level (Figure 1B). (*S*)-FTY720 vinylphosphonate induces the proteasomal degradation of SK1b in LNCaP-AI cells (Figure 2A). Differences in the sensitivity of SK1b to (*S*)-FTY720 vinylphosphonate in terms of proteasomal degradation in LNCaP and LNCaP-AI cells might suggest that SK1b is differentially post-translationally modified in the two cell types, and that this affects responsiveness to (*S*)-FTY720 vinylphosphonate, although this requires further investigation.

### 2.3. Differential Effect of (R)- and (S)-FTY720 Vinylphosphonate on S1P Levels in Human Lung Endothelial Cells

The substantial increase in S1P levels in both cell types (Figure 1(A,B)) suggests that (*S*)-FTY720 vinylphosphonate might also inhibit S1P lyase and/or S1P phosphatase activity. In this regard, it is noted that FTY720 is a weak inhibitor of S1P lyase [20]. (*S*)-FTY720 vinylphosphonate does not activate SK2 [13], and therefore the increase in intracellular S1P is unlikely to occur via this mechanism. Therefore, we assessed the effect of (*S*)-FTY720 vinylphosphonate on the uptake and metabolism of sphingosine in human lung endothelial cells to more directly assess whether (*S*)-FTY720 vinylphosphonate can regulate the formation of S1P. Sphingosine is taken up into these cells and converted to S1P by SK1, although the amount of S1P that accumulates is subject to metabolism by S1P lyase and S1P phosphatase. The data displayed in Table 2A, B show that (*S*)-FTY720 vinylphosphonate at 3 and 24 h increased the amount of [^32^P]S1P that accumulates in these cells in response to sphingosine, suggesting that (*S*)-FTY720 vinylphosphonate might also act as an S1P lyase or S1P phosphatase inhibitor. In contrast, (*R*)-FTY720 vinylphosphonate, which is also an SK1 inhibitor [8], reduced [^32^P]S1P accumulation at 3 and 24 h in response to sphingosine (Table 2(A,B)). The possible differential inhibitory activity of the enantiomers of FTY720 vinylphosphonate on S1P lyase and S1P phosphatase will be assessed in future experiments.

**Table 2:** The effect of FTY720 vinylphosphonate on S1P formation from sphingosine in human lung endothelial cells. [^32^P]-labeled cells were treated with sphingosine (2 μM) for 30 min. Cells were preteated with either 10 μM (*S*)-FTY720 vinylphosphonate ((*S*)-vinylPn) or 10 μM (*R*)-FTY720 vinylphosphonate ((*R*)-vinylPn) for (**A**) 3 h or (**B**) 24 h. The [^32^P] S1P formed is normalized to 100,000 dpm in total lipid extracts. Results are means +/− SD for n = 3 samples.

**(A)**	[^32^P] **S1P formed (dpm)**
Vehicle	878 +/− 6
Sphingosine	4025 +/− 47
(*S*)-vinylPn + Vehicle	1124 +/− 58
(*S*)-vinylPn + Sphingosine	5150 +/− 18
(*R*)-vinylPn + Vehicle	640 +/− 40
(*R*)-vinylPn + Sphingosine	2326 +/− 32
**(B)**	[^32^P] **S1P formed (dpm)**
Vehicle	1015 +/− 69
Sphingosine	3665 +/− 264
(*S*)-vinylPn + Vehicle	1011 +/− 35
(*S*)-vinylPn + Sphingosine	5530 +/− 358
(*R*)-vinylPn + Vehicle	724 +/− 84
(*R*)-vinylPn + Sphingosine	2627 +/− 178

We have previously shown that (*S*)-FTY720 vinylphosphonate induces apoptosis as assessed by the increased cleavage of polyADPribose polymerase (PARP), which is a DNA repair enzyme, in LNCaP-AI cells [8]. We now show that this is associated with an increase in C16:0-ceramide. However, (*S*)-FTY720 vinylphosphonate also induces apoptosis of LNCaP cells (Figure 2B), where ceramide levels remain unaltered at 24 h (Figure 1B). In this case, high levels of S1P might contribute to the apoptosis of LNCaP cells. Indeed, there is evidence that very high levels of S1P are pro-apoptotic in other cell types, such as endothelial cells [21]. In addition, S1P and (*E*)-2-hexadecenal can cooperate with BAK/BAX to induce the permeabilization of the outer mitochondria membrane, which results in the release of cytochrome c, an early step in intrinsic apoptosis [22]. Therefore, the apoptotic effect of (*S*)-FTY720 vinylphosphonate might be promoted by the substantial increase in intracellular S1P in LNCaP cells. In contrast, the apoptotic effect of (*S*)-FTY720 vinylphosphonate might be promoted by high intracellular S1P and C16:0-ceramide levels in LNCaP-AI cells. The effect of SKi and (*S*)-FTY720 vinylphosphonate on C22:0- and C16:0-ceramide levels in LNCaP and LNCaP-AI cells, respectively, might also suggest the involvement of different ceramide synthase isoforms linked with SK1 in these different cell types.

### 2.4. Effect of (S)-FTY720 Vinylphosphonate on the Metabolome of LNCaP and LNCaP-AI Cells

The levels of Ap3A do not change in LNCaP cells treated with (*S*)-FTY70 vinylphosphonate ((*S*)-vinylPn/control 0.86, P = 0.6, n = 3) and was undetectable in LNCaP-AI cells. Moreover, treatment of LNCaP and LNCaP-AI cells with (*S*)-FTY70 vinylphosphonate failed to induce changes in the metabolome that would indicate oxidative stress ((*S*)-lactoylglutathione levels actually decreased in LNCaP cells) or antagonism of the Warburg effect (data not shown). Therefore, these effects are different compared with SKi. Moreover, the metabolites that were modestly changed in response to (*S*)-FTY70 vinylphosphonate were phospholipids, such as lysophosphatidylethanolamine (lyso-PE), phosphatidylserine (PS), and phosphatidic acid (PA) in LNCaP-AI cells (Table 3). These changes were less evident in LNCaP cells (Table 3). The change in PS might be linked with apoptosis of LNCaP-AI cells, indicating distinct mechanisms of apoptosis in response to (*S*)-FTY720 vinylphosphonate in the cell types. However, (*S*)-FTY70 vinylphosphonate, SKi, and ROME also induce changes in lyso-PE (Table 3), indicating that these inhibitors might modulate some common pathways. 

Table 3Effect of (**A**) (*S*)-FTY720 vinylphosphonate (10 μM, 24 h) on metabolite levels in LNCaP-AI and LNCaP cells; (**B**) ROME (10 μM, 24 h) on lyso-PE levels in LNCaP cells; (**C**) SKi (10 μM, 24 h) on lyso-PE levels in LNCaP cells. Results are expressed as the ratio inhibitor treated:control for each metabolite. P values are presented for inhibitor *versus* control (experimental results from n = 3 cell samples for each treatment). PA, phosphatidic acid; PS, phosphatidylserine; lyso-PE, lysophosphatidylethanolamine, ND, not detected

**(A):** 

Compound	Ratio (*S*)-vinylPn- treated/controlLNCaP-AI	P value	Ratio (*S*)-vinylPn- treated/controlLNCaP	P value
**Lipids**				
PA 32:1	2.39	< 0.01	1.35	0.36
PA 32:2	2.50	< 0.001	2.07	< 0.05
PA 34:2	2.33	< 0.001	1.54	0.11
PS 32:1	2.04	< 0.02		
PS 38:6	2.16	< 0.01	ND	
Lyso-PE 18:1	2.18	< 0.01	0.92	0.61
Lyso-PE 20:1	2.10	< 0.01	1.02	0.85
Lyso-PE 20:2	2.10	< 0.01	0.97	0.78
**Oxidative stress**				
GSH	0.98	0.76	0.75	< 0.01
S-D-Lactoylglutathione	0.97	0.83	0.61	< 0.01
GSSG	0.88	0.39	0.58	0.06

**(B):** 

Compound	Ratio ROME- treated/control LNCaP	P value
Lyso-PE 18:0	1.53	< 0.05
Lyso-PE 18:1	1.36	< 0.05
Lyso-PE 20:1	1.61	< 0.05

**(C):** 

Compound	Ratio SKi-treated/control LNCaP	P value
Lyso-PE 18:0	3.0	< 0.01
Lyso-PE 18:1	1.8	< 0.01

**Figure 3:**
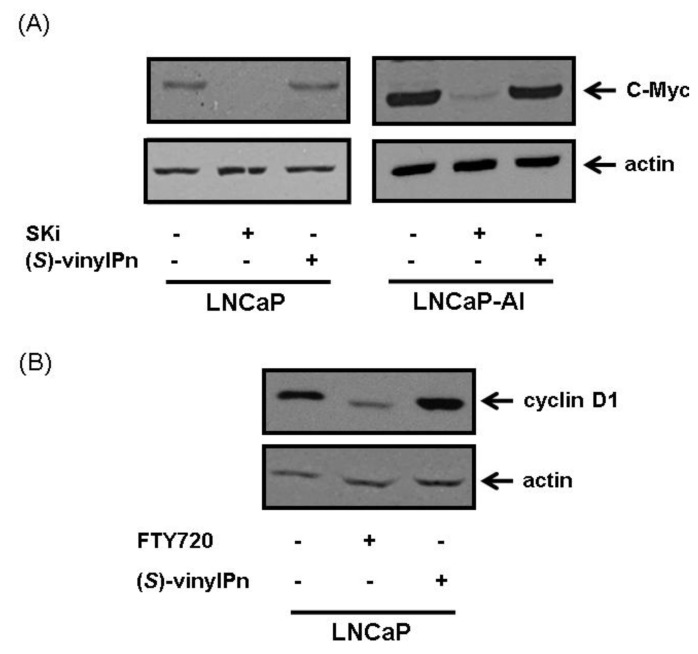
Effect of (**A**) SKi and (*S*)-FTY720 vinylphosphonate on c-Myc expression in LNCaP and LNCaP-AI cells. Cells were treated with (*S*)-FTY720 vinylphosphonate (50 μM) or SKi (50 μM) with vehicle (DMSO, 0.1% v/v) alone for 18 h. (**B**) FTY720 and (*S*)-FTY720 vinylphosphonate on cyclin D1 expression in LNCaP cells. Cells were treated with FTY720 (10 μM, used a positive control) or (*S*)-FTY720 vinylphosphonate (10 μM) or vehicle (DMSO, 0.1% v/v) alone for 18 h. The lysates were subjected to western blot analysis with anti-cyclin D1 or actin antibodies. Results are representative of at least 3 separate experiments. (*S*)-vinylPn represents (*S*)-FTY720 vinylphosphonate.

The lack of effect of (*S*)-FTY70 vinylphosphonate on glycolytic metabolites and oxidative stress are distinct from those observed for SKi, even though both compounds are able to induce proteasomal degradation of SK1. The major reason for this might relate to the extent to which these compounds inhibit SK1 activity in cells. We have shown that SKi activates the proteasome via a ceramide-dependent mechanism that appears contingent on the inhibition of SK1 activity by SKi [7]. The activation of the proteasome also causes enhanced degradation of c-Myc and cyclin D1 in response to SKi at concentrations ranging from 10 to 50 μM (Figure 3(A,B)) [7,10]. Therefore, the effects of SKi on Warburg effect might be accounted for, by changes in c-Myc expression, which is a master transcriptional regulator of glycolytic enzymes. However, (*S*)-FTY720 vinylphosphonate at concentrations as high as 50 μM failed to induce degradation of c-Myc and cyclin D1 in LNCaP and LNCAP-AI cells (Figure 3(A,B)), indicating that this compound does not activate the proteasome. Therefore, (*S*)-FTY720 vinylphosphonate is unlikely to affect aerobic glycolysis, oxidative stress and Ap3A formation. It is, therefore, important to understand how SKi and (*S*)-FTY720 vinylphosphonate exert differential effects on the proteasome, yet both are able to induce proteasomal degradation of SK1. It is possible that (*S*)-FTY720 vinylphosphonate binds to SK1 to induce a conformation that exhibits enhanced sensitivity to proteasomal degradation. In contrast, SKi enhances the rate of SK1 degradation by activating the proteasome [7]. In this regard, it is significant that (*S*)-FTY720 vinylphosphonate is an allosteric inhibitor of SK1, while SKi is predominantly a competitive inhibitor (with sphingosine) [9,11] and (*S*)-FTY720 vinylphosphonate might therefore induce a conformation of the enzyme that is susceptible to enhanced polyubiquitination and proteasomal degradation. It will be necessary to establish in the future the impact of catalytic inhibition *versus * proteasomal degradation of SK1 on different metabolic pathways in prostate cancer cells, some of which might be regulated by the removal of c-Myc.

### 2.5. Effect of ROME on the Metabolome of LNCaP Cells

To further investigate the function of SK2, and to discriminate between the effects of SK1- *vs.* SK2-selective inhibitors, we assessed the effect of the SK2-selective inhibitor ROME on the metabolome of LNCaP cells. We have previously shown that the inhibition of SK2 with ROME increases sphingosine and reduces S1P levels with no effect on ceramide (10). The data in Table 4 show that treatment of LNCaP cells with ROME substantially increased the levels of several 1-ester-2-lysophosphatidylinositols (lyso-PI) and modestly increased 1-ester-2-lysophosphatidic acid (LPA) species. In particular, there is a 218-fold increase in 1-palmitoyl-2-lyso-PI levels with ROME. These findings provide the first evidence to indicate the presence of a possible cross-talk between SK2 and lyso-PI metabolism in cancer cells. Both lyso-PI and LPA are ligands for GPR55 and LPA/EDG receptors that promote proliferation and cell survival [23,24]. Therefore, these metabolite changes are consistent with the possibility that SK2 normally functions to limit mitogenic signaling. 

**Table 4:** Effect of ROME (10 μM, 24 h) on lyso-PI and LPA species in androgen-sensitive LNCaP cells. Results are expressed as the ratio ROME:control for each metabolite. P values are presented for ROME *vs.* control (experimental results from n = 3 cell samples for each treatment).

	Ratio of ROME : control	P value n = 3
Lyso-PI 16:0	218.2	< 0.005
Lyso-PI 18:0	30.0	< 0.005
Lyso-PI 18:1	22.2	< 0.005
Palmityl glucuronide	11.2	< 0.01
LPA 16:0	2.4	< 0.05
LPA 16:1	1.4	< 0.01
LPA 18:0	3.6	< 0.05

The glucuronide conjugate of palmityl alcohol (Table 4) is also elevated in ROME-treated cells. This metabolite might be an intermediate derived from the conversion of S1P into (*E*)-2-hexadecenal and phosphoethanolamine by S1P lyase. (*E*)-2-Hexadecenal is likely to be rapidly converted to either palmitic acid or hexadecanol. The question then is how might inhibition of SK2 and lowering of S1P increase the levels of the glucuronide conjugate of hexadecanol? We have previously shown that SK2 is involved in rapid cycling of S1P and sphingosine, which appears to preclude access of S1P to S1P lyase [10]. 

Inhibition of SK2 might prevent sphingosine-S1P recycling if the S1P phosphatase activity is regulated by substrate availability. Therefore, in the absence of functional SK2 activity in ROME-treated cells, the availability of S1P for the phosphatase might be reduced. Under these conditions, S1P might become available to the S1P lyase. This could result in a reduction in S1P levels and the formation of the glucuronide conjugate of hexadecanol that is observed in ROME-treated cells. Indeed, others have shown that inhibition of the S1P phosphatase decreases S1P levels, and it has been proposed that under these conditions, S1P becomes accessible to S1P lyase [25]. Long-chain alcohols tend to be conjugated to glucuronide to aid excretion, and small amounts of this conjugate were detected in the cell growth medium in response to ROME (data not shown). Taken together, these findings provide additional evidence that SK2 has predominantly opposing action to SK1 in androgen-sensitive LNCaP cells, which might involve restriction of mitogenic signaling pathways. Moreover, we have previously shown that SKi inhibits autophagy (10, 15), while ROME stimulates autophagy (15).

## 3. Experimental Section

### 3.1. Materials

All general biochemicals and anti-actin antibody were from Sigma (Poole, UK). RPMI 1640, penicillin-streptomycin (10000 U/mL penicillin and 10000 μg/mL streptomycin), and L-glutamine were from Invitrogen (Paisley, UK). European fetal calf serum (EFCS) was purchased from Sera Laboratories (Haywards Heath, UK). Charcoal filtered fetal bovine serum was from Lonza (Switzerland). SKi was from Merck Biosciences (Nottingham, UK). Anti-PARP/cleaved PARP antibody was purchased from New England Biolabs Ltd. (Hitchin, UK), anti-actin antibody was from Sigma (Poole, UK) and anti-c-Myc and anti-cyclin D1 antibodies were from Santa Cruz (California, USA). ROME was synthesized as described previously [13]. LNCaP and LNCaP-AI cells were gifts from Professor Hing Leung (Beatson Institute, Glasgow). Anti-SK1b antibody was a kind gift from Professor A. Huwiler (University of Berne, Switzerland).

### 3.2. Cell Culture

Human prostate cancer LNCaP and LNCaP-AI cell lines were maintained in RPMI 1640 medium supplemented with 10% EFCS or 10% delipidated serum, respectively, 1% penicillin-streptomycin, and 1% L-glutamine. All cells were maintained in a humidified atmosphere at 37 °C with 5% CO_2_. Cells were treated with inhibitors or vehicle for 24–48 h, as indicated for each experiment. When cells were treated for 48 h, the inhibitor or vehicle was replaced after 24 h. LNCaP-AI cells are derived by culturing LNCaP cells in androgen-deprivation conditions [26]. AR receptor is still expressed in androgen-independent LNCaP-AI cells, but growth and proliferation are androgen-independent [25]. Human lung microvascular endothelial cells (HLMVECs), passages between 5 and 8, were grown to contact-inhibited monolayers with typical cobblestone morphology in EGM-2 complete media with 10% FBS, 100 U/mL penicillin and streptomycin in a 37 °C incubator under 5% CO_2_-95% air atmosphere [27]. Cells from T-75 flasks were detached with 0.05% trypsin and resuspended in fresh complete medium and cultured in 35-mm dishes.

### 3.3. Preparation of Whole Cell Extracts

Cell extracts for SDS-PAGE and Western blot analysis were prepared by washing treated cells with 5 mL of PBS and then re-suspending the cell pellets in whole cell lysis buffer [(137 mM NaCl, 2.7 mM KCl, 1 mM MgCl_2_, 1 mM CaCl_2_, 1% v/v NP40, 10% v/v glycerol, 20 mM Tris) (pH 8.0) containing 0.2 mM PMSF, 10 μg/mL leupeptin, 10 μg/mL aprotinin, 0.5 mM Na_3_VO_4_, 100 µM NaF, and 10 mM β-glycerophosphate]. Samples were repeatedly (x6) passed through a 23-gauge needle using a syringe and rotated for 30 min at 4 °C to allow for efficient lysis. Cell debris was pelleted by centrifugation at 22000 × *g* for 10 min at 4 °C, and the supernatant (whole cell extract) was collected. The protein content was measured using the Pierce BCA assay kit (Fisher Scientific, Loughborough, UK). For each sample, 10–20 μg of protein was added to sample buffer [125 mM Tris, pH 6.7, 0.5 mM Na_4_P_2_O_7_, 1.25 mM EDTA, 0.5% w/v SDS containing 1.25% v/v glycerol, 0.06% w/v bromophenol blue, and 50 mM dithiothreitol], and used for SDS-PAGE and western blotting using anti-actin, anti-SK1b, anti-Myc, anti-cyclin D1 and anti-PARP antibodies. 

### 3.4. Measurement of Intracellular [^32^P]S1P Generation

HLMVECs (~90% confluence) in 35-mm dishes were labeled with [^32^P]orthophosphate (20 µC_i _/ mL) in phosphate-free DMEM media for 3h in the presence or absence of (*S*)-FTY720- or (*R*)-FTY720-vinylphosphonate (10 μM) conjugated to 0.1% BSA. In other experiments, cells were pretreated with (*S*)-FTY720- or (*R*)-FTY720-vinylphosphonate (10 μM) overnight prior to labeling with [^32^P]orthophosphate (20 µC_i _/ mL) in phosphate-free DMEM media for 3 h. Cells were challenged with sphingosine (2 µM) in the presence of 0.1% BSA for 30 min. The radioactive medium was aspirated and lipid labeling was terminated by addition of 1 mL of methanolic HCl (methanol:HCl ratio 100:1, v/v) and 1 mL of 1M HCl. Cells were harvested with a cell scraper, the total extract was transferred to 15 mL glass tubes, lipids were partitioned after vortexing into the chloroform phase by addition of 2 mL of chloroform and 800 µL of 1M HCl (to give a final ratio of 1: 1: 0.9 of chloroform:methanol:acidic aqueous phase). After vortexing, the lower (chloroform) phase was dried under nitrogen and the lipid extracts were subjected to thin-layer chromatography (TLC). Lipid extracts were applied at 10 cm from the bottom of 20-cm plastic-baked silica gel 60 plates. The plate was developed in chloroform/methanol/NH_4_OH (65:35:5, v/v/v), air dried for 20 min, and then cut 2.0 cm above the origin. This removed neutral lipids and most of the zwitterionic phospholipids while several acidic phospholipids such as PA, S1P, LPA, and ceramide 1-phosphate remained near the origin. The top part of the cut plate was discarded and the bottom of the plate was then developed in the reverse direction with chloroform/methanol/glacial acetic acid/acetone/water (10:2:3:4:1, v/v/v/v). Dried plates were subjected to autoradiography; the area corresponding to labeled S1P was excised and radioactivity was determined by liquid scintillation counting. The data were normalized to total radioactivity in the lipid extract [28].

### 3.5. Analysis of Sphingoid Base 1-Phosphates and Ceramides

Analyses of sphingoid base 1-phosphates, ceramides, and sphingoid bases were performed by electrospray ionization tandem mass spectrometry (ESI-LC/MS/MS) on an AB Sciex 5500 QTRAP hybrid triple quadrupole linear ion-trap mass spectrometers (Applied Biosystems, Foster City, CA) equipped with a TurboIonSpray ionization source interfaced with an automated Agilent 1200 series liquid chromatography (Agilent Technologies, Wilmington, DE). S1P and dihydro-S1P (DHS1P) were analyzed as bis-*O*-acetylated derivatives with C17-S1P as the internal standard employing reverse-phase HPLC separation, negative ion ESI, and Multiple Reaction Monitoring (MRM) analysis as described in [29]. Ceramides and dihydroceramides were analyzed using C17:0-ceramide as an internal standard with reverse-phase HPLC separation, positive ion ESI, and MRM analysis as described in [30]. Sphingoid bases were analyzed with C17-sphingosine as the internal standard, positive ion ESI, and MRM as described in [30].

### 3.6. Isolation of Cell Extracts and Liquid Chromatography Mass Spectrometry

Cells (1 × 10^6^) were plated in T-25 cell culture flasks and grown until the cell number doubled (48 h) before being treated with SKi (10 μM) or (*S*)-FTY720 vinylphosphonate (10 μM) or ROME (10 μM) or vehicle for 24 h. Cell extracts were prepared by washing the cells twice with PBS at 37 °C before harvesting the cells into pre-cooled extraction solution (MeOH/MeCN/H_2_O, 50:30:20) (1 mL per 2 × 10^6 ^cells). Cell lysates were mixed at 4 °C at 1440 rpm for 12 min before being centrifuged at 0°C at 13000 rpm for 15 min. The supernatants were collected and transferred into HPLC vials for loading into the LC-MS autosampler. The chromatographic conditions were as follows: A ZICpHILIC column (150 × 4.6 mm × 5 µm) was eluted with a linear gradient over 30 min between 20 mM (NH_4_)_2_CO_3_ (pH 9.2)/MeCN (20:80) at 0 min and 20 mM (NH_4_)_2_CO_3 _(pH 9.2)/MeCN (20:80) at 30 min with a flow rate of 0.3 mL/min, followed by washing with 20 mM (NH_4_)_2_CO_3_ (pH 9.2)/MeCN (95:5) for 5 min and then re-equilibration with the starting conditions for 10 min. LC/MS was carried out by using an Accela HPLC pump coupled to an Exactive (Orbitrap) mass spectrometer from Thermo Fisher Scientific (Bremen, Germany). The spray voltage was 4.5 kV for positive mode and 4.0 kV for negative mode. The temperature of the ion transfer capillary was 275 °C and sheath and auxiliary gas was 50 and 17 arbitrary units, respectively. The full scan range was 75 to 1200 *m/z* for both positive and negative modes. The data were recorded using the Xcalibur 2.1.0 software package (Thermo Fisher Scientific). The signals of 83.0604 m/z (2xACN+H) and 91.0037 *m/z* (2 × formate-H) were selected as lock masses for the positive and negative modes, respectively, during each analytical run. 

### 3.7. Data Extraction

Data extraction was carried out by using Sieve 1.3. The ion chromatograms were pasted into an Excel macro written in house and the library was searched against a database of accurate masses for compounds in the Human Metabolome Data Base, KEGG, and Metlin. 

## 4. Conclusions

The major effects of SKi, (*S*)-FTY720 vinylphosphonate and ROME are summarized in Table 5. Our findings using SK inhibitors suggest that SK1 can regulate aerobic glycolysis, Ap3A formation, and apoptosis of androgen-sensitive LNCaP cells, and that the ability of SK1b to evade the proteasome in response to SKi in androgen-independent LNCaP-AI cells might be linked with aberrant Ap3A formation, thereby promoting cell survival in the presence of SKi. We have also suggested that SK1 might be linked with different ceramide synthases in LNCaP and LNCaP-AI cells, such that removal of SK1 from these cells has differential effects on C22:0- and C16:0-ceramide metabolism. In addition, we demonstrate that SK2 might functionally regulate lyso-PI and LPA metabolism possibly linked with mitogenesis. Therefore, SK2 appears to have a non-overlapping function compared with SK1 and is likely to regulate a different pool of S1P in prostate cancer cells. The inhibition of SK1, SK2, S1P phosphatase and S1P lyase by these compounds, which might be localized in different sub-cellular compartments in prostate cancer cells, could result in distinct cellular regulation of the metabolome, thereby influencing unique cellular responses. This consideration is therefore worthy of further study in terms of improving our understanding of how these enzymes are involved in controlling apoptosis of prostate cancer cells. For instance, the proteasomal degradation effect of SK1 in response to (*S*)-FTY720 vinylphosphonate [13], allied to a stimulatory effect on S1P formation, suggests that (*S*)-FTY720 vinylphosphonate might inhibit S1P phosphatase and/or S1P lyase that are functionally coupled with SK2 and not SK1. This results in a substantial increase in a sub-cellular pool of S1P that might promote apoptosis via a mechanism that is distinct from that induced by down-regulation of SK1 in response to SKi. In the latter case, SK1 appears to function by removing apoptotic ceramide that might be localized in a different compartment compared with SK2, and linked to apoptosis via regulation of the proteasome; this being dependent on a ‘threshold activity’ of SK1.

**Table 5:** Summary of effects of SKi, (*S*)-FTY720 vinylphosphonate, and (*R*)-FTY720 methyl ether (ROME) on prostate cancer cells.

Compound	SKi	(*S*)-FTY720 -vinylPn)	ROME
**Inhibitory specificity**	SK1/2	SK1 selective	SK2 selective
**Proteasome**	Activator	No effect	No effect
**SK1 expression**	Proteasomal degradation of SK1a/SK1b in LNCaP but only SK1a in LNCaP-AI	Proteasomal degradation of SK1a/SK1b in LNCaP-AI	No effect on SK1 expression
**Apoptosis**	Inducer in LNCaP No effect in LNCaP-AI	Inducer in LNCaP and LNCaP-AI	No effect
**Authophagy**	Inhibition	ND	Stimulation
**Oxidative stress**	Induction	No effect	No effect
**Glycolysis**	Increased glycolytic metabolites; reduced flux?	No effect	Reduced glycolytic metabolites: increased flux?
**c-Myc expression**	Reduced	No effect	No effect
**Androgen receptor expression**	Reduced	Reduced	No effect
**Lipid metabolism**	Lyso-PE modestly increased in LNCaP	Lyso-PE modestly increased in LNCaP-AI	Lyso-PI substantially increased in LNCaP
